# Oxidative Stress in Non-alcoholic Fatty Liver Disease. An Updated Mini Review

**DOI:** 10.3389/fmed.2021.595371

**Published:** 2021-02-26

**Authors:** Anna Pia Delli Bovi, Francesca Marciano, Claudia Mandato, Maria Anna Siano, Marcella Savoia, Pietro Vajro

**Affiliations:** ^1^Pediatrics Section, Department of Medicine and Surgery, Scuola Medica Salernitana, University of Salerno, Baronissi, Italy; ^2^Department of Molecular Medicine and Medical Biotechnologies, University of Naples Federico II, Naples, Italy; ^3^Department of Pediatrics, Santobono-Pausilipon Children's Hospital, Naples, Italy

**Keywords:** non-alcoholic fatty liver disease, oxidative stress, antioxidants, obstructive sleep apnea syndrome, gut microbiota, obesity, metabolic syndrome

## Abstract

Non-alcoholic fatty liver disease (NAFLD) is a challenging disease caused by multiple factors, which may partly explain why it remains still orphan of an adequate therapeutic strategy. Herein we focus on the interplay between oxidative stress (OS) and the other causal pathogenetic factors. Different reactive oxygen species (ROS) generators contribute to NAFLD inflammatory and fibrotic progression, which is quite strictly linked to the lipotoxic liver injury from fatty acids and/or a wide variety of their biologically active metabolites in the context of either a two-hit or a (more recent) multiple parallel hits theory. An antioxidant defense system is usually able to protect hepatic cells from damaging effects caused by ROS, including those produced into the gastrointestinal tract, i.e., by-products generated by usual cellular metabolic processes, normal or dysbiotic microbiota, and/or diet through an enhanced gut–liver axis. Oxidative stress originating from the imbalance between ROS generation and antioxidant defenses is under the influence of individual genetic and epigenetic factors as well. Healthy diet and physical activity have been shown to be effective on NAFLD also with antioxidant mechanisms, but compliance to these lifestyles is very low. Among several considered antioxidants, vitamin E has been particularly studied; however, data are still contradictory. Some studies with natural polyphenols proposed for NAFLD prevention and treatment are encouraging. Probiotics, prebiotics, diet, or fecal microbiota transplantation represent new therapeutic approaches targeting the gut microbiota dysbiosis. In the near future, precision medicine taking into consideration genetic or environmental epigenetic risk factors will likely assist in further selecting the treatment that could work best for a specific patient.

## Introduction

The term *non-alcoholic fatty liver disease* (NAFLD) was originally coined by Ludwig et al. (1). It indicated a hepatopathy similar to that of alcohol abuse without alcohol consumption history, and it is now reputed as the hepatic component of metabolic syndrome (2, 3). It affects approximately a quarter of the population, mostly obese, and has no approved drug therapy. Although NAFLD is generally benign, ~20–30% of patients develop liver inflammation, fibrosis/cirrhosis (non-alcoholic steatohepatitis, NASH), and, in some cases, hepatocellular carcinoma (4, 5). Moreover, patients with NAFLD are at higher risk of cardiovascular diseases. Because of the lack of valid therapies and of the obesity pandemic, NAFLD is one of rapidly growing indications for liver transplantation (6).

Most NAFLD patients are obese and present a mild systemic inflammation, which hampers insulin signaling [insulin resistance (IR)], playing a relevant role in the pathomechanism of liver damage (7, 8). Recently, in consideration of this association, an international group of experts highlighted the poor coherence of the term *non-alcoholic fatty liver disease* and proposed that of *metabolic (dysfunction)–associated fatty liver disease* (9). The reason why some patients with simple steatosis show a progression to more severe hepatic injury, whereas others do not, was in part simplified by the so-called “two-hit” model, founded on IR, and the deposits of relatively inert triglycerides (TGs) within the liver as initial damage. This first event was thought to be due to a “second hit” generated by oxidative stress (OS) or depletion of ATP (10) with the activation of an inflammatory cytokine cascade contributing to the development of NASH necroinflammation and fibrosis (10–12).

**Figure 1:**
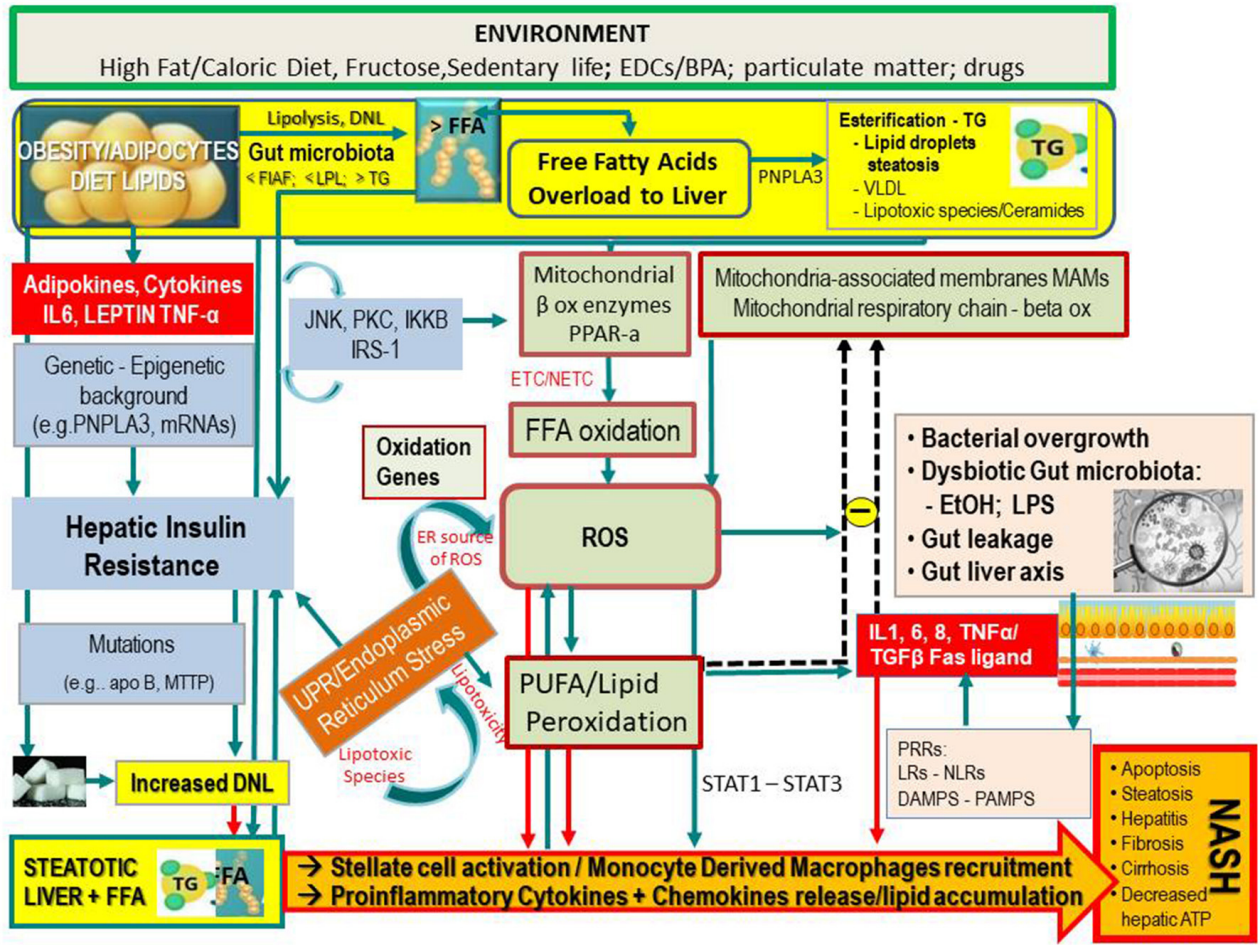
Simplified flow of pathogenetic events in non-alcoholic fatty liver disease. The figure shows the crosstalk between systems and metabolisms in the pathogenetic events leading to fatty liver and its progression to NASH. In the upper part (yellow lane), one can note that hepatic FFAs derive from lipolysis in adipose tissue, dietary lipids, and DNL from COH in the liver. These FFAs may either be stored in the liver as TG droplets (hepatic steatosis) or be exported as VLDL to adipose tissue. FFA overload may concur in the hepatic IR (vertical light azur lane), which interplays with the JNK, PKC system where the activation of JNK1 may impair insulin signaling via serine phosphorylation of IRS1. The UPR/ER stress is a source of ROS and of lipotoxic species and plays a link between the OX stress and IR. Upon disruption of mitochondria-associated membranes (MAM) integrity, miscommunication directly or indirectly disrupts Ca^2+^ homeostasis and increases ERS (brown box) and OS, leading to defective insulin secretion and accelerated lipid droplet formation in hepatocytes. Inflammatory mediators (adipokines, cytokines) in large part arrange the progression from NAFLD to NASH (red boxes) in case of shortage of endogenous antioxidant molecules. These mediators are variously triggered by oxidative hepatic environment [ROS, lipid peroxidation] and bacterial overgrowth (pink boxes) after the infraction of the gut barrier (gut leakage) by bacterial Eth and enhanced intestinal permeability, which allows lipopolysaccharides (a) to activate PRR–LRs—NLRs–DAMPS—PAMPS and (b) to concur with ROS/PUFA in the inhibition of the mitochondrial respiratory chain. Lipotoxic lipid species lead to hepatic stress and subsequent release of extracellular vesicles, cytokines, chemokines, and DAMPs from hepatocytes. This results in enrolment of bone marrow immune cells. As shown in the lower part of the figure, liver-resident stellate/KCs are activated by several triggers (mainly ROS, gut microbiota), resulting in the release of chemokine (C-C motif) ligand 2 (CCL2) and other proinflammatory cytokines (i.e., TNF-α, IL-1, and IL-6). The oxidative hepatic environment also stimulates transcription programs (STAT-1 and STAT-3) promoting T-cell recruitment and hepatic disease progression. Overall, the scenario ultimately leads to the recruitment of bone marrow–derived monocytes and neutrophils that further contribute to the inflammatory response and a rebound ROS production. A number of genetic variants are implicated in NAFLD development, and progression is shown. BPA, bisphenol A; CYP, cytochrome; COH, carbohydrates; DAMPS, damage-associated molecular patterns; DNL, *de novo* lipogenesis; EDC, endocrine-disrupting chemicals; ETC, electron transport chain; Eth, ethanol; FFA, free fatty acids; FIAF, fasting-induced adipose factor; HNE, hydroxynonenal; IKKB, inhibitor of nuclear factor κB kinase subunit β; IL, interleukin; IRS, insulin receptor substrate; JNK, c-Jun N-terminal kinase; LPL, lipoprotein lipase; LPS, lipopolysaccharide; LRs, lectin receptors; MAM, mitochondria-associated membrane; MDA, malondialdehyde; mRNA, microRNA; MTTP, microsomal triglyceride transfer protein; NAFLD, non-alcoholic fatty liver disease; NASH, non-alcoholic steatohepatitis; NLRs, NOD-like receptors; NF-κB, nuclear factor κ-light-chain enhancer of activated B cells; NTC non-electron transport chain; PAMPs, pathogen-associated molecular patterns; PKC, protein kinase; PNPLA3, patatin-like phospholipase domain-containing protein 3; PRR, pattern recognition receptor; PPAR, peroxisome proliferator-activated receptor; PUFA, polyunsaturated fatty acids; ROS, reactive oxygen species; TG, triglyceride; TNF, tumor necrosis factor; UPR, unfolded protein response; VLDL, very low-density lipoprotein; <, decrease; >, increase.

However, it has been found that hepatic lipid accumulation in NAFLD occurs mostly as relatively inert TGs droplets, and this is nowadays regarded as a protective rather than a deleterious mechanism, by impeding the storage of free fatty acids (FFAs), which are the actual harmful agents in this hepatopathy. Most recent evidences underline that inflammation may even precede fat accumulation, which would become only a response (12, 13). As schematically shown in Figure 1, hepatic FFAs originate from lipolysis in adipose tissue and dietary lipids. Moreover, particularly in conditions of IR, they may also be synthesized *de novo* (so-called *de novo* lipogenesis) from carbohydrates in the liver and be deposited as TG droplets (hepatic steatosis), or exported contributing to the very low-density lipoprotein pool (14).

The previous “two-hit theory” has therefore led the way to the “multiple parallel hits theory” (12), with the contribution of a number of “multiple parallel (and not sequential)” offenders acting with different combinations, at times synergistically, to generate NAFLD. These offenders include, in addition to IR (3) and OS, hormones secreted from the adipose tissue, intestinal dysbiosis, increased intestinal permeability, and also exposure to environmental agents such as endocrine disruptors (15) and particulate matter (PM) (16, 17) interacting among themselves in individuals predisposed by genetic and epigenetic factors.

Genes that modulate hepatic fat accumulation and retinol metabolism [i.e., transmembrane 6 superfamily member 2 (TM6SF2), variants of patatin-like phospholipase domain which contain protein 3 (PNPLA3), membrane-bound O-acyltransferase domain containing 7 (MBOAT7), hydroxysteroid 17β-dehydrogenase (HSD17B13), and glucokinase regulator (GCKR)] (9) and the deregulation of microRNAs are known to influence NAFLD development and progression (18).

In addition, also genetic variants involved in OS regulation play an important role in NAFLD pathogenesis. These genes include SOD2 gene, coding for the manganese-dependent superoxide dismutase (MnSOD); UCP3, coding for the uncoupling protein 3, a mitochondrial transporter that enhances the proton leak of mitochondrial inner membrane and unhooks the oxidative phosphorylation; uncoupling protein 2 (UCP2), regulating oxidative metabolism and mitochondrial lipid efflux; and MARC1 (A165T), which codes for the mitochondrial amidoxime reducing component 1, a protein involved in the neutralization of reactive oxygen species (ROS) (19, 20).

The NAFLD story is even more complex than this, as it may start even before conception and pregnancy. Epigenetic changes, comprising microRNA features, may cause fetal reprogramming during the pregnancy of an obese mother and transgenerational transmission of the susceptibility to NAFLD in childhood and progression to NASH across the lifetime. Moreover, improving obese mothers' diet reduces fetal hypoxemia and counteracts metabolic pathways able to generate OS, liver injury precursors, and lipotoxicity in non-human primates (21–23).

On the basis of the most recent literature, herein we will focus especially on OS because the understanding of a main role for OS in NAFLD development and progression can have important preventive and therapeutic implications for possible novel treatments.

## Oxidative Stress and Its Role in NAFLD Pathology

OS is caused by a discrepancy between ROS generation and antioxidant defenses, which lead to DNA and tissue damage (24, 25). It may occur both for the increasing production of pro-oxidant products and the dysfunction of the antioxidant system.

Although it is essential to tissue repair, it may conceal also negative features implying the development and/or exacerbation of several systemic diseases and conditions [e.g., mental/neurological diseases (26, 27), inflammatory bowel diseases (28), cardiovascular disease (29), and cancer (30)]. Starting from these premises, one can therefore easily predict that OS represents an important mediator triggering low-grade inflammation also in metabolic syndrome and in the progression of NAFLD into NASH (31–35).

ROS, in fact, appear tightly involved in those processes that lead to hepatic fibrosis (36). Multiple interlaced pro-oxidative triggers operate together with the mitochondrial dysfunction as a likely common denominator of OS (37). In NASH, there are more evidences of mitochondrial DNA and protein abnormalities being responsible for the increase of OS (38, 39). A decreased oxidative capacity of the electron transport chain (ETC) and mutations in complex II could also lead to a condition of “electron leakage” (40), meaning that the electron normal flow could be interrupted, binding with oxygen to produce superoxide or hydrogen peroxide. Moreover, the levels of glutathione (GSH) peroxidase, MnSOD, and catalase seem to be low in NASH, so that the capability of the mitochondria to reduce ROS levels is reduced. In NASH patients, an increased activity of CYP2E1 (41) has been also observed, an important microsomal source of OS, especially together with C47T polymorphisms of SOD2 (encoding MnSOD) (41–45).

In the development of NASH, OS probably occurs not only due to the saturation of the antioxidant machinery secondary to the increased pro-oxidant species production and its direct insult. In the liver, actually, these conditions trigger lipid peroxidation by specific polyunsaturated fatty acids (PUFAs), along with the formation of highly reactive aldehyde products [e.g., malondialdehyde (MDA) and 4-hydroxy-2-non-enal (4-HNE)]. Overall, these events appear involved in the diffusion of ROS and reactive nitrogen species (RNS) into the extracellular space, perpetuating intracellular and tissue damage. Moreover, hepatic OS may result from gut microbiota (GM)–related inflammation and the disturbance in the normal functions of endoplasmic reticulum [so-called ER stress (ERS)] (see below) [(37, 46); Figure 1].

## Implication of the Oxidative Stress in Hepatic Injury

ROS/RNS (i.e., hydrogen peroxide, superoxide anion radical, peroxynitrite, and hydroxyl radical), and not lipid peroxidation byproducts, are the responsible for cytokine elevations (47) such as tumor necrosis factor α (TNF-α), transforming growth factor β, interleukin 8 (IL-8), and Fas ligand. The sum of these events results in NAFLD development (25). The oxidative hepatic environment in obesity furthermore promotes the signal transduction and activation of transcription programs (STAT-1 and STAT-3) that promote T-cell recruitment and liver damage with disease progression up to its malignant transformation [(48); Figure 1].

### OS and Hepatic Injury: Possible Implications in NAFLD Progression

Hepatocyte damage involves a cascade of events leading to NAFLD progression into NASH and cirrhosis: damage-associated molecular patterns, discharged from damaged hepatocytes, lead to the release of chemokines and cytokines from Kupffer cells (KCs) and the recruitment of monocyte-derived macrophages. ROS directly and indirectly contribute to stellate cell activation and to chronic inflammatory response with up-regulation of proinflammatory cytokines (TNF-α, IL-6, and IL-1), apoptosis, and development of hepatic fibrosis [(49–51); Figure 1].

In conditions of progressive NAFLD, OS can also result from increased ROS generation due to impairment of mitochondria caused by an overload of FFAs and an increase of their metabolism, lipotoxicity, and hypoxia, as well as ROS production through NADPH-oxidase isoforms associated to ligand–receptor link or by activated inflammatory cells (49).

Evidences suggest that lipotoxicity mediated by FFAs (52) may induce disruption of ER homeostasis, known as “unfolded protein response,” an intracellular signaling activated by the accumulation of unfolded/misfolded proteins. Thanks to it, ER can communicate the folding status of its proteins to the rest of the cell, particularly to the nucleus, and so activate genes transcription. As a result, the ERS, a term that includes also several other mechanisms conducing to ROS generation, occurs (37), and this leads to

- increased endoplasmic reticulum oxidoreduction-1 (ERO-1) activity, the enzyme that catalyzes disulfide bond formation (53) with H_2_O_2_ production;
- upregulation of CCAAT/enhancer-binding protein homologous protein (Chop), a proapoptotic mechanism (54);
- calcium leakage from ER, which increases its flow through mitochondrial membranes leading to proapoptotic mitochondrial membrane permeabilization (55);
- GSH depletion (56), altering GSH–oxidized glutathione balance, which is essential to redox homeostasis; and
- inhibition of nuclear factor, erythroid 2–related factor 2, a factor encoding for antioxidant proteins (57).

The cross talk between ERS and ROS (Figure 1) appears relevant in the pathogenesis of NAFLD (58). Mitochondria-associated membranes (MAMs) represent a physical junction between ER and mitochondria, allowing Ca^2+^, lipids, and ROS exchange. Because normal communication between mitochondria and ER depends on MAM structural and functional integrity, lack of calcium homeostasis may lead to ERS and OS increase, defective insulin secretion, and accelerated lipid droplet formation in hepatocytes. The steps involve apoB misfolding, impaired lipoprotein secretion, and lipogenesis stimulation. On these bases, protecting the ER via the administration of antioxidants or activation of peroxisome proliferator-activated receptor (PPAR) has been suggested as promising avenues against hepatic steatosis (59, 60).

Studies in rodents show the existence of a link between ERS and regulation of hepatic iron metabolism both in ASH and NASH models mainly due to the capacity of ferrous iron to catalyze the production of hydroxyl radical (OH^−^) from H_2_O_2_, deriving by peroxisomal β-oxidation (52, 61, 62). Interestingly, iron deficiency too may reduce the cell antioxidant capability by inhibiting heme oxygenase-1 by Bach1 (63). Beyond doubt, it is not always simple to study the progression of a disease, especially in humans *in vivo*, and establish if a certain factor is exactly the cause or the effect of NASH. Moreover, a disagreement often happens between animal models and clinical studies due to several factors such as gut microflora differences and patient inclusion criteria/ethnicity–related predisposition, respectively (37).

In order to assess the redox state in NAFLD/NASH, some markers of OS and antioxidants have been studied in NAFLD and NASH models, both clinical and experimental. OS biomarkers include nitric oxide, lipid damage products (lipid peroxides, thiobarbituric acid reactive substances/MDA), hydroperoxides, 8-isoprostane, 4-HNE, DNA oxidation product [CYP2E1 and 8-hydroxydeoxyguanosine (8-OH-dG)], and protein oxidation products (nitrotyrosine, protein carbonyl). All these had increased activities in most NAFLD/NASH clinical models evaluated. On the contrary, antioxidant markers (superoxide dismutase, catalase, glutathione peroxidase, reduced glutathione) measured in rodent models showed decreased activities mainly in NASH (64).

## Gut Microbiota and Intestine Permeability as a Cause of Oxidative Stress

### Gut Microbiota as a Source of ROS

Human commensal microbiota (Figure 1) generates physiological ROS levels in intestinal human epithelial cells. Basically, aerobic cell systems are exposed to oxygen free radicals (65, 66), and their damaging role relies on their concentrations. When the levels of ROS exceed antioxidant defenses, harmful effects on cells may occur, conducing to uncontrolled proliferation, inflammation, and/or apoptosis (67, 68). This is what happens also in obesity and its related hepatometabolic comorbidities, including NAFLD progression to NASH (see below). ROS can also operate as second messengers in intracellular signaling stimulated by proinflammatory cytokines and growth factors and by the quick and reversible oxidative inactivation of proteins having thiol groups sensitive to oxidants (69). In inflammation and obesity, ROS generation is probably strictly related with activation of nuclear factor κ-light-chain enhancer of activated B cells (NF-κB) and degradation of NF-κB inhibitor (IκB), making NF-κB more transcriptionally active (70–72). As shown in Figure 1, a quantitative or qualitative (in term of dysbiosis) bacterial alteration (small intestine bacterial overgrowth) is also concatenated with OS through the inhibition of mitochondrial respiratory chain.

### Interaction Between Gut Microbiota, OS, and Intestinal Permeability in NAFLD

Gut mucosal barrier separates, functionally and physically, the luminal content from the underlying compartment that, in addition to gut epithelia, includes immune, vascular, and structural elements in the lamina propria. The intestinal mucosa is constantly exposed to oxidants and carcinogens taken in from diet and/or bacteria, whose chronic exposure may cause production of free radicals leading to redox imbalance and subsequent DNA damage, disturbing the intestinal metabolic equilibrium (73).

GM plays an important role in different processes (metabolic, nutritional, physiological, and immunological) involved in maintaining a healthy status (69, 74). Its qualitative and quantitative composition differs in the distinct parts of gastrointestinal (GI) tract because of the influences by different conditions [e.g., age, dietary habits, ethnicity, delivery mode, exposure to therapies, pathogens, and contact with several environmental stimuli (75–78)]. Perturbation of GM composition, called “dysbiosis,” has been recognized in diseases associated not only with the GI tract [e.g., inflammatory bowel disease (79)] but also with systemic conditions such as obesity, diabetes mellitus, autism, depression, and NAFLD (80). While a quite clear causal role of a specific GM has been demonstrated in murine models of NAFLD (e.g., unhealthy diet dependent shift from Bacteroidetes to Firmicutes), recognition of a corresponding human microbiome signature is more difficult. In fact, it may be hindered by the components of associated metabolic syndrome and several other confounding factors. Anyway, Gram-negative harmful bacteria release lipopolysaccharide (LPS), lipoteichoic acid, flagellin, lipoprotein, or other toxins recognized by the pattern recognition receptors (PRRs) expressed on the surface of innate immune system cells. Similarly, structurally conserved motifs present on the surface of different types of pathogens (pathogen-associated molecular patterns) are recognized and bound by PRRs, inducing mitochondrial ROS production and nuclear gene expression.

PRR classes sensitive to the microbiota's factors are Toll-like receptor (TLR), Rig-1-like receptor, Nod-like receptor, and C-type lectin receptor. They induce the NF-κB pathway activation and enhance the inflammatory response when proinflammatory cytokines and antibacterial factors are released (81, 82). Differently, small formylated peptides produced and released by commensal bacteria are recognized by another kind of receptor, known as formylated peptide receptors. These are G-proteins linked to surface receptors of neutrophils and macrophages, stimulating ROS synthesis in phagocytes and epithelial cells (83). In particular, their activation stimulates superoxide anion production by NADPH oxidase 1, increasing ROS levels in cell cytoplasm that lead to an inflammatory response and increase of cell OS (84). As a consequence of cell stress, mitochondrial and bacterial DNA may be integrated in the nuclear genome causing the alteration of cellular gene expression.

Intestinal mucosa permeability has an important role in modulating how GM can influence also other parts of the body. An alteration of its barrier function consents to the GM and its endotoxins to cross the intestinal epithelium and the endothelial barrier (85) traveling into systemic circulation and reaching different target organs (75, 86).

There are many evidences according to which gut bacteria are involved in the pathogenesis of liver injury induced by alcohol, and gut leakiness promotes proinflammatory bacterial products reaching the liver, thus initiating the proinflammatory cascade that causes alcoholic steatohepatitis (ASH). Alcohol impairs intestinal epithelial cell permeability *in vitro* through a mechanism mediated by OS (87), supporting therefore the idea that OS may be the main cause of alcohol-induced intestinal leakage (88, 89).

In NAFLD, with a quite similar mechanism, endogenous ethanol produced by some microbial species [e.g., *Escherichia* genus members of the Proteobacteria phylum induced by high-fat diet; (90)] is able to induce the formation of ROS by HSC cells and impair intestinal integrity. The latter allows LPS to reach hepatic TLRs activating and further enhancing oxidative, inflammatory, and fibrogenetic mechanisms (75, 90, 91, 91–94).

GM seems to mediate the progression from simple steatosis to NASH. In particular, increased Gram-negative bacteria expose KCs to an elevated amount of LPS and upregulation of PRRs (37). It has been hypothesized that the endocytosis of LPS by KC could induce upregulation of cytokine receptors, especially the TNF-α receptor, which seems to be also involved in the increased ROS production (95). Activated KCs have a role in IR, fibrosis development, and inflammation amplification.

**Figure 2:**
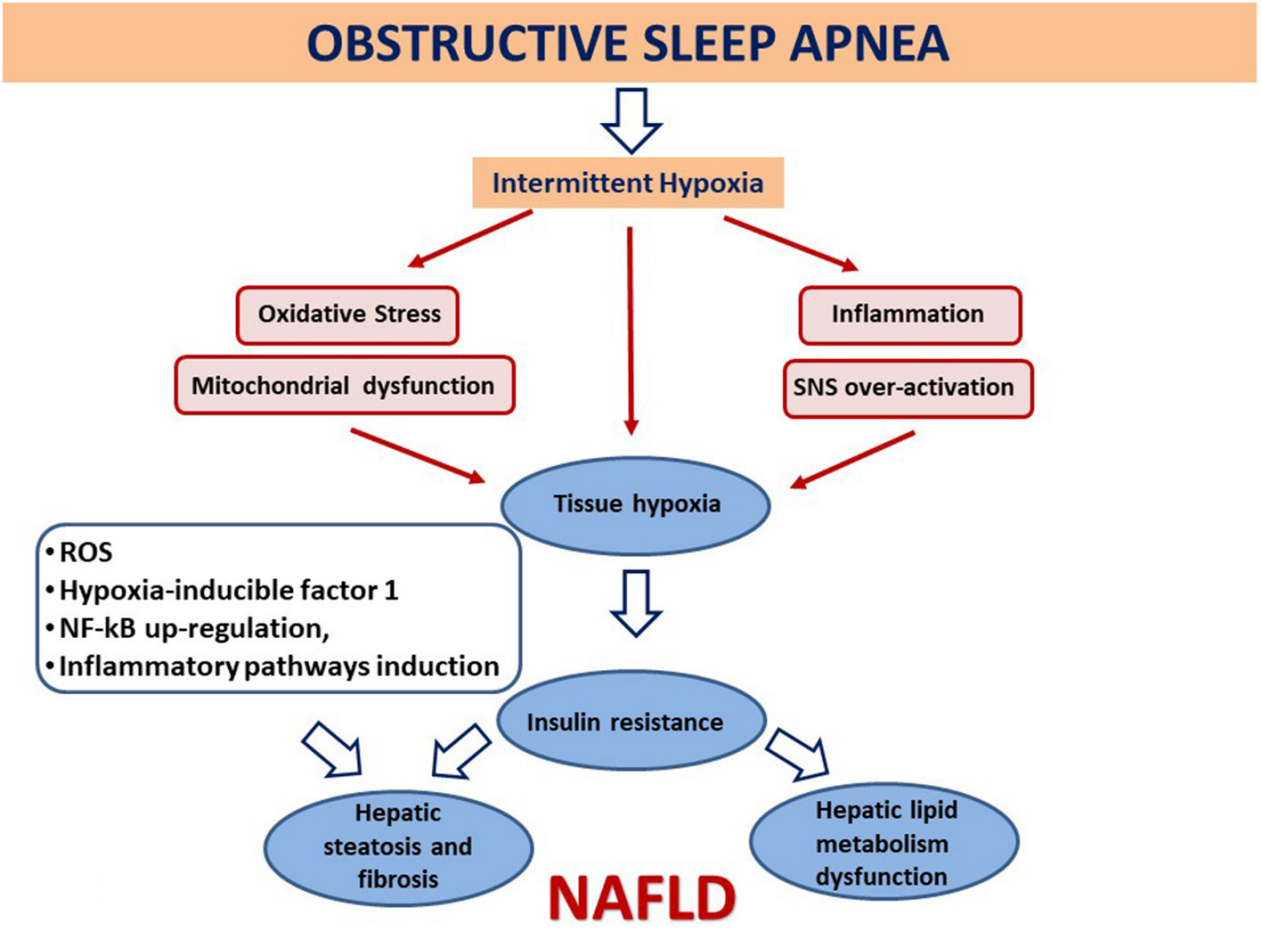
Association between obstructive sleep apnea and the development and evolution of non-alcoholic fatty liver disease. Intermittent hypoxia leads to tissue hypoxia, OS, mitochondrial dysfunction, inflammation, and overactivation of the sympathetic nervous system (SNS). Generated reactive O_2_ species (ROS) may amplify liver injury by activating hypoxia-inducible factor 1, a transcriptional activator and master regulator of O_2_ homeostasis during hypoxia, and by up-regulating nuclear factor κ-light-chain enhancer of activated B cells (NF-κB), with subsequent downstream induction of inflammatory pathways. As a consequence, this involves insulin resistance, dysfunction of key steps in hepatic lipid metabolism, atherosclerosis, and hepatic steatosis and fibrosis, each of which is pertinent to the development and/or progression of non-alcoholic fatty liver disease (NAFLD) (98–101).

Also, the association between obstructive sleep apnea syndrome (OSAS) and NASH severity seems to correlate with endotoxemia increase and gut barrier function alteration, conducing to increased hepatic susceptibility to endotoxemia mediated by TLR-4 (96). An alarming 60% OSAS incidence has been reported in pediatric NAFLD (97, 98). This disorder of breathing during sleep has been associated with fatty acid accumulation in the liver and inflammation caused by frequent nocturnal hypoxia (NH), IR, OS, and adipokine dysregulation (99). Growing experimental evidences link the alternation of NH with normoxia (so-called chronic intermittent hypoxia) caused by OSAS to NAFLD development and progression (49, 100). A study that compared healthy controls and NAFLD patients (some of which with OSA/NH), identified NH as a possible source of OS in NAFLD. OSA/NH is common in pediatric patients with liver biopsy-proven NAFLD and is associated with more advanced liver injury and histological disease (97, 98). Intermittent hypoxia conduces to tissue hypoxia and can lead to OS, mitochondrial malfunction, inflammation, and sympathetic nervous system hyperactivation. As a consequence, intermittent hypoxia causes IR, impairment of hepatic lipid metabolism pathways (84), and hepatic steatosis and fibrosis, each of which is involved into NAFLD development and/or progression [(101); Figure 2].

## Therapeutic Strategies

### Non-enzymatic Anti-oxidants Defenses

Antioxidants are substances that inhibit the oxidation of any biomolecule (102), neutralizing the harmful effects of oxidation caused by free radicals, maintaining therefore the redox homeostasis. Antioxidants are either synthesized endogenously (e.g., GSH, superoxide dismutase) or taken from the diet. Anthocyanins, lycopene, coenzyme Q10, flavonoids, β-carotene, lipoic acid, selenium, lutein, catechins, and vitamins A, C, and E are among the many substances normally present in foods that possess a high antioxidant activity. As reported in the table, they can be also classified in two large groups on the basis of the presence/absence of their enzymatic action [(103–114); Table 1].

**Table 1:** Antioxidants with and without enzymatic action.

**Enzymatic antioxidants**	**Non-enzymatic antioxidants**
Superoxide dismutase (SOD)	**Low-molecular-weight compounds** Glutathione, thioredoxin, lactoferrin
	**Endogenous substances** Lipoic acid, melatonin, albumin, bilirubin, uric acid, polyunsaturated fatty acids omega 3
Catalase (CAT)	**Flavonoid polyphenols** Silymarin (104, 105) Blueberry leaf, bergamot polyphenols (115, 116)
	**Stilbenes** Resveratrol (106, 107)
Glutathione peroxidase	**Herbs** Erchen decoction, danshen, berberine (108)
	**Carotenoids** β-Carotene, astaxanthin, lycopene, β- cryptoxanthin, lutein, fucoxanthin, crocetin (109, 110)
Paraoxonase 1 (PON 1)	**Phenolic compounds** Açai (111)
	**Vitamins** Ascorbic acid (vitamin C), α-tocopherol [vitamin E, vitamin A, vitamin D (112–114)]

Despite the above premises, antioxidants as potential pharmacological agents have hitherto not appeared extremely effective *in vivo* as either a preventive or therapeutic tool in NAFLD (31, 117–122). Studies that have investigated the role of vitamin E as a treatment of NASH confirm that it acts against pathogenic mechanisms conducting to liver damage and NASH, thanks to its antioxidant and anti-inflammatory activity (123–126). The antioxidant power of vitamin E is due to the hydroxyl group in the tocochromanol ring, which neutralizes free radicals and ROS by donating hydrogen. The major forms of tocopherol and tocotrienol are α, β-, γ-, and δ-, with the antioxidant activity of the δ-isoform being weaker than the others, the vitamin E isoforms are also involved in many other activities (Table 2). Among the vitamin E isoforms, the α-tocopherol, has other different properties independently from its antioxidant ability: it can inhibit the activity of protein kinase C, reducing the proliferation of different cell types (vascular smooth muscle cells, mesangial cells, neutrophils, monocytes/macrophages, fibroblast, and various cancer cell lines) and the 5-lipoxygenase pathway, inhibiting the release of proinflammatory cytokine IL-1β.

**Table 2:** Activities influenced by vitamin E isoforms.

I. Regulation of the inflammatory response
II. Gene expression
III. Membrane-bound enzymes
IV. Cellular signaling
V. Cell proliferation
VI. Regulation of several enzymes involved in signal transduction: Protein kinase C (PKC), protein phosphate 2A (PP2A), 5-lipoxygenase, cyclooxygenase 2 (COX-2), and monocyte chemoattractant protein 1 (MCP-1)
VII. Regulation of several factors in the mitogen-activated protein kinase (MAPK) signal transduction pathway

### Clinical Trials of Vitamin E for NAFLD: Vitamin E in the Clinics

Available data are still conflicting. The largest trials with vitamin E in NAFLD are the PIVENS (Pioglitazone vs. Vitamin E vs. Placebo for the Treatment of Non-diabetic Patients with Non-alcoholic Steatohepatitis) (127) and the TONIC (Treatment of Non-alcoholic Fatty Liver Disease in Children) (121) trials. The first showed that both drugs tested in adults ameliorated steatosis, lobular inflammation, and hepatocellular ballooning, but did not ameliorate fibrosis. Vitamin E but not pioglitazone induced a clinical improvement in NASH. The TONIC trial, which evaluated therapeutic intervention with vitamin E vs. metformin in children with NAFLD, showed that both improved hepatocellular ballooning and the NAFLD activity score (NAS), but neither vitamin E nor metformin decreased alanine aminotransferase (ALT) values or hepatic steatosis, inflammation, or fibrosis in NASH. The reasons of these disappointing results depend on the need of better patient selection and protocols. Interestingly, a most recent systematic review and meta-analysis (1,317 patients from 15 randomized controlled trials) concluded that vitamin E improves biochemical and histological outcomes in adults and pediatric patients, with a significant negative association between transaminases levels and vitamin E dosage—more satisfactorily ranging between 400 and 800 IU. However, while adults receiving vitamin E improved significantly transaminases, fibrosis, and NAS both at early and late follow-up, children showed more significant changes at long-term follow-up, which could partly explain the negative results obtained by certain short-term studies (128). Some still unsolved safety concerns should be considered as well. Vitamin E, in fact, has been suspected to have a dichotomous suppressive and promoting activity with respect to tumorigenesis [e.g., co-cancerogenic in prostate cancer; (25, 129)] possibly explainable by still poorly studied host gene–supplement interactions (130). In our opinion, further carefully designed studies are still necessary for substantiating this view and supporting optimum procedures in terms of both efficacy and safety profiles.

Results from multiple regression models showed a significant negative association between ALT, AST levels, and vitamin E dosage—more favorably between 400 and 800 IU.

**Figure 3:**
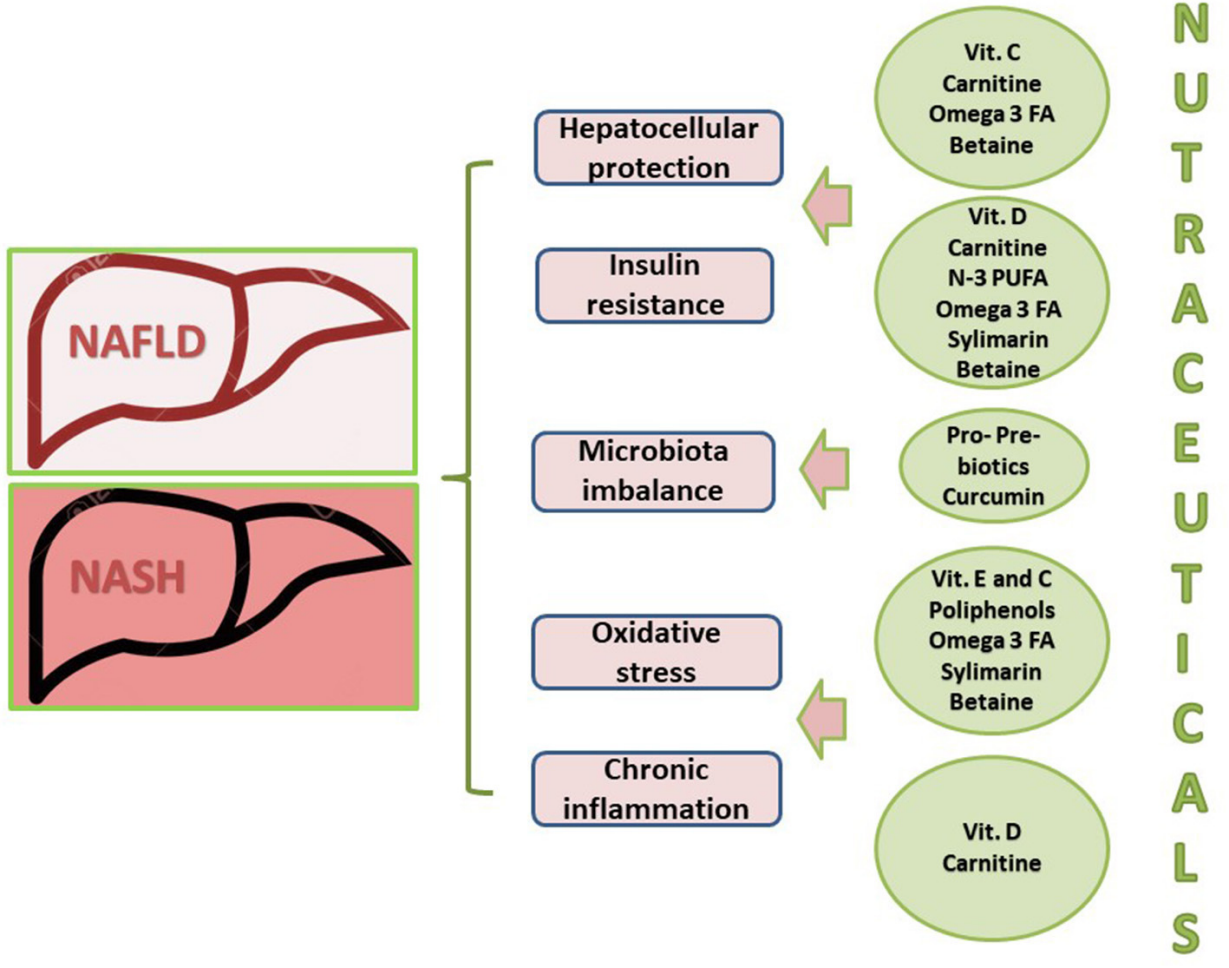
Multiple targets of nutraceuticals for the treatment of non-alcoholic fatty liver disease. FA, fatty acids; NAFLD, non-alcoholic fatty liver disease; NASH, non-alcoholic steatohepatitis. Adapted and modified by Del Ben et al. (103).

A quite large number of other nutraceutical antioxidants that seem to improve NASH through more than one pathway (Table 2 and Figure 3) include but are not limited to the following:

- Curcumin (37, 103), with effects on different amino acids, bile acids, tricarboxylic acid cycle, and GM (131, 132), although only few human clinical trials are available (132–134).
- PUFAs of omega-3 series (PUFA omega-3), which may act as an antioxidant, have a role in modulating OS improving the defense capacity against an increased oxidative burden (135, 136).

Thanks to them, the cellular metabolism switch from lipogenesis and triacylglycerol accumulation to fatty acid oxidation thus plays a role in decreasing fatty liver. Furthermore, they have anti-inflammatory activity and enhance insulin sensitivity (103). Clinical trials evaluating the efficacy of n-3 PUFA (including docosahexaenoic acid and eicosapentaenoic acid) on systemic OS in NAFLD and NASH have shown controversial results. While n-3 PUFA supplementation appears useful in NAFLD early stages (137), unfortunately, total (enzymatic and non-enzymatic) antioxidant capacity is not enough to attenuate the hepatic damage (35). Interestingly, dietary antioxidant intake is significantly lesser in NASH patients than in healthy controls (138).

- Among polyphenols, blueberry leaf polyphenols appear to have a positive effect on hepatic mitochondrial dysfunction and redox homeostasis, whereas bergamot polyphenolic formulation seems to improve IR, hepatocellular ballooning, inflammation, and fibrosis (115, 116).

### Probiotics and Prebiotics: Other Tools Improving Defenses Against OS

Improving defenses against OS through modulation of the GM composition and functionality offers a promising means of managing or treating metabolic disorders (74).

Probiotics are living microorganisms with beneficial health activity on the host. For example, they are able to improve GM composition and reduce LPS serum amount and liver TLR4, delaying liver disease progression (81, 98, 99). Lactobacilli and bifidobacteria are the most commonly used, usually present in dietary supplements or fermented foods such as yogurt and cultured milk (100). Changing the resident GM composition and the gut lumen, they create an anti-inflammatory environment, obtaining decreased proinflammatory bacterial products and gut barrier integrity improvement. *Lactobacillus rhamnosus* GG (LGG) is the subject of numerous studies (139–144); it has different beneficial effects on the intestinal function through dysbiotic microbiota normalization (100, 144, 145) and reducing intestinal OS (146). A recent meta-analysis found a beneficial effect of probiotics also on hepatic antioxidative capacity as mirrored by the increase of SOD and GSH-PX activities and decrease of MDA content (147). A daily LGG treatment in alcohol-fed rats significantly improves severity of ASH and gut leakiness induced by alcohol, decreases intestinal and liver OS markers and inflammation, and normalizes the gut barrier task, avoiding to trigger liver disease (148). GM regulates also the powerful antioxidant glutathione and amino acid metabolism (144). It is not surprising therefore that fecal microbiota transplantation (FMT) from control donors in steatotic rats has been found to have beneficial effects in terms of decrease of portal hypertension through insulin sensitivity improvement mediated by the endothelial nitric oxide synthase signaling pathway, a pathway clearly involved in the antioxidant mechanisms (149). A pilot study of FMT in NASH is currently undergoing to evaluate whether restoration of healthful GM through FMT from lean donors (FMT-L) ameliorates NASH (150).

Similarly, “prebiotics” are fermentable carbohydrates that selectively modulate microbiota composition and/or activity, resulting in a beneficial effect for the host (146). Finally, also synbiotics (i.e., a combination of prebiotics and probiotics) have shown a positive effect on GM and have been proposed as a support for the treatment of NAFLD (151).

Summing up, the modulation of quality and diversity of every single human microbiota appears therefore an appealing tool in the management of intestinal ROS, OS, inflammation, and some metabolic anomalies caused by dysbiosis (152). Moreover, it is suggested that changes in GM occurring upon prebiotic consumption may be due to gut bacterial functions improvement. In other words, products generated by *Lactobacillus* and metabolites derived by microbiota, such as antioxidants and fatty acids, could be employed for target medicine in the management of liver disease including NAFLD (146).

## Other Therapeutic Strategies

### Drugs

Ursodeoxycholic acid (UDCA) remains one of the most studied drugs: in addition to exerting a possible therapeutic effect on NAFLD by modulating autophagy and apoptosis dysregulation, UDCA appears to have also antioxidant properties (153).

A number of other drugs that have been tested for their influence on hepatic steatosis have still uncertain/elusive molecular mechanisms. There are several innovative agents currently undergoing phases II and III clinical trials with different targets (154).

Obeticholic acid, a semisynthetic bile acid analog, is an agonist of the farnesoid X receptor, which has anti-inflammatory and antioxidant activities (155).

Silymarin, a botanical product extracted from milk thistle, because of its antioxidant properties appears to improve NAFLD hypertransaminasemia and reduce liver disease progression in NASH, but at present, available results are inconclusive (156).

Cannabidiol, a chemical without psychotropic effects, has antioxidant and anti-inflammatory properties by acting on the endocannabinoid system. After stimulation of the G-protein–coupled receptors and their endogenous lipid ligands, it interferes with progression toward NASH (26, 157).

### Physical Activity

Physical activity (PA) acts favorably in NAFLD primarily by reducing intrahepatic fat content with β-oxidation of fatty acids and lipogenesis regulation, enhancing the expression and activity of PPAR-γ, insulin sensitivity, and hepatoprotective autophagy, reducing hepatocyte apoptosis, and inflammation of the liver by decreasing the proinflammatory mediators. PA, moreover, has several beneficial effects on NAFLD also with the improvement of several antioxidants activity [e.g., catalase, SOD, glutathione peroxidase and reductase, glutathione-S-transferase, thioredoxin reductases, NADH cytochrome B5 reductase, and NAD(P)H quinone acceptor oxidoreductase], leading to decreased ROS production and proinflammatory cytokines (158).

## Concluding Remarks

Our review exhibits that OS not counteracted by intact antioxidant defense system plays an important role in NAFLD/NASH with a number of other casual factors. Excessive FFA β-oxidation due to increased FFA fueling leads to excessive ROS formation, which, in turn, downregulates ETC, and non-ETC systems, affect insulin sensitivity, hepatic lipid metabolism, and inflammatory responses by interacting with innate immune signaling (159).

Gut dysbiosis may induce further signaling processes, which engage the epithelium and immune/inflammatory cells. In these conditions, GM may take advantage of the increased intestinal permeability and/or impairment of epithelial tight junctions. This results in an enhancement of the gut–liver axis with bacteria and endotoxin transit through the intestinal and endothelial vascular wall, ending up into hepatic and other systemic diseases as well.

The above scenario would suggest a therapeutic role of antioxidants in patients with fatty liver disease, but this approach has not been entirely translated yet in human (160), as most studies still derive from murine models with substantial differences in genetic background and in the digestive system; the need to perform more human studies appear evident (161).

Vitamin E has shown promising data but without significant benefit in fibrosis improvement (162). Several natural polyphenols and n-3 PUFA supplementation provided with a number of antioxidant, antiobesity, and anti-inflammatory effects could have potential in NAFLD prevention and treatment by acting on its multifactorial pathogenetic components, but also here data either to support or refuse their use are insufficient (115, 116).

In addition to healthy diet (e.g., a Mediterranean diet seems to reduce OS), probiotics, prebiotics, and fecal transplantation appear to be emerging strategies to modulate microbiota quality and diversity, in order to prevent and/or avoid gut damage. Avoidance of exposure to endocrine disruptors (15) and to ambient PM (16, 17) also appears strategic to add benefices to NAFLD.

Last but not least, the accurate assessment of NAFLD-associated genetic/epigenetic risk factors of diseases and likelihood of disease progression is going to aid to target individualized appropriate treatments (163).

## Author Contributions

FM and APDB collected literature and prepared the first draft of the manuscript. MS and MAS collected literature on specific areas and gave critical suggestions. PV gave critical suggestions, made substantial intellectual contributions to the study design, and manuscript preparation. CM provided a major intellectual input, verified/contributed to data analysis, and took over writing of the manuscript as and when required. All authors gave substantial contributions to the work and revised critically and approved the final manuscript.

## Conflict of Interest

The authors declare that the research was conducted in the absence of any commercial or financial relationships that could be construed as a potential conflict of interest.
